# Mouse Models for Pendrin-Associated Loss of Cochlear and Vestibular Function

**DOI:** 10.1159/000356635

**Published:** 2013-12-18

**Authors:** Philine Wangemann

**Affiliations:** Anatomy & Physiology Department, Kansas State University, Manhattan, Kansas, USA

**Keywords:** *Slc26a4*, Enlarged vestibular aqueduct, Hearing, Cochlea, Endolymphatic sac, Genetic disease model

## Abstract

The human gene *SLC26A4* and the mouse ortholog *Slc26a4* code for the protein pendrin, which is an anion exchanger expressed in apical membranes of selected epithelia. In the inner ear, pendrin is expressed in the cochlea, the vestibular labyrinth and the endolymphatic sac. Loss-of-function and hypo-functional mutations cause an enlargement of the vestibular aqueduct (EVA) and sensorineural hearing loss. The relatively high prevalence of *SLC26A4* mutations provides a strong imperative to develop rational interventions that delay, ameliorate or prevent pendrin-associated loss of cochlear and vestibular function. This review summarizes recent studies in mouse models that have been developed to delineate the role of pendrin in the physiology of hearing and balance and that have brought forward the concept that a temporally and spatially limited therapy may be sufficient to secure a life-time of normal hearing in children bearing mutations of *SLC26A4*.

## Introduction

The gene *SLC26A4* (MIM #605646) codes for the protein pendrin, which is an electroneutral exchanger for anions such as HCO_3_^−^, Cl^−^, and I^−^ [1–3]. Pendrin is predominantly expressed in epithelial cells of the inner ear, the thyroid, and the kidney but has also been found in airways, mammary gland and liver [4–10].

The expression of *SLC26A4* in the inner ear and the thyroid is consistent with the observation that mutations of *SLC26A4* (MIM #605646) cause congenital hearing loss associated with an enlarged vestibular aqueduct (EVA; MIM #600791) and Mondini-like dysplasia of the cochlea and enlargement of the thyroid (Pendred syndrome; MIM #274600). Hearing loss is typically progressive, often fluctuating, and occurs with an onset before or around the time of speech and language acquisition [11, 12]. Vestibular deficits are less overt [13–16].

The prevalence and the spectra of *SLC26A4* mutations vary among populations. Mutations have been reported to occur in 5.6%, 5–10%, or 13.7% of children with congenital hearing loss [17–19]. The relatively high prevalence provides an imperative to investigate the etiology of *SLC26A4*-related deafness with the ultimate goal to develop strategies to preserve hearing in affected individuals.

This review provides a brief summary of recent studies in mouse models that have been developed to delineate the role of pendrin in the physiology of hearing and balance. Clinical phenotypes, the genetics of hearing loss associated with an enlargement of the vestibular aqueduct and more details on the development of the murine inner ear are reviewed elsewhere [20–22].

## Development of the murine inner ear

The development of the inner ear begins in mice at embryonic day 9.5 (E9.5) with the formation of an otocyst [23]. The otocyst encloses amniotic fluid, which is a plasma-like fluid containing ~140 mM Na^+^ and ~10 mM K^+^ [24]. Between E10 and E10.5, two protrusions begin to extend from the otocyst; one forms the cochlea and the other forms the endolymphatic sac. While the protrusions elongate, the center of the otocyst reorganizes into the vestibular labyrinth. The lumen of the endolymphatic sac opens at E10.5 and the lumen of the cochlea opens at E14.5 [25]. Lumen formation during the growth phase of the inner ear is controlled by a balance of fluid secretion and absorption. Fluid secretion appears to occur in the vestibular labyrinth and fluid absorption in the endolymphatic sac [25]. The hypothesis that Cl^−^ secretion and Na^+^ absorption control luminal volume during the growth phase of the inner ear is consistent with the finding that the epithelial lumen of the cochlea and the endolymphatic sac is filled until E17.5 with a solution that contains ~140 mM Na^+^, ~126 mM Cl^−^, ~10 mM K^+^, and ~25 mM HCO_3_^−^ [26].

At E17.5 the interconnected fluid compartments of the inner ear separate into two systems [27]. One system consists of the utricle, three ampullae and three semicircular canals and the other of the cochlea, the saccule and the endolymphatic sac. Vestibular sensory cells acquire mechanosensitivity at E17 [28, 29]. The onset of vestibular function is ~8 days later, at postnatal day 4 (P4), concurrent with the general maturation of the organ and the maturation of the innervation [30, 31]. Cochlear sensory cells acquire mature mechanosensitivity at P0 [29]. The onset of hearing is ~12 days later, at P12, concurrent with the development of the endocochlear potential, which rises between P5 and P15 from ~10 mV to the mature voltage of ~90 mV [32–34].

With the conclusion of the growth phase of the cochlea, the luminal fluid, endolymph, changes from a NaCl to a KCl solution. The onset of K^+^ secretion is at E19.5 [26]. Two days later, at postnatal day 0, cochlear endolymph already contains ~70 mM K^+^ and 5 days later, at P3, endolymph contains ~100 mM K^+^, which is close to the mature concentration of 150 mM K^+^ [26]. The rise of the K^+^ concentration is paralleled by a decline of the Na^+^ concentration [26]. In the mature inner ear, the Na^+^ concentration amounts to 1 mM and the Ca^2+^ concentration to 22 μM, both concentrations being unusually low for an extracellular fluid [35].

## Pendrin expression in the inner ear

In the mature inner ear, pendrin is expressed in the apical membrane of non-sensory epithelial cells in the cochlea, the vestibular labyrinth and the endolymphatic sac [6, 36]. During development, the earliest expression in the inner ear occurs at E11.5 in the endolymphatic sac [37]. Between E13.5 and E14.5, expression in the endolymphatic sac surges dramatically, and virtually all pendrin expression in the E14.5 inner ear is located in the endolymphatic sac. In the cochlea, the earliest expression of pendrin is found in the hook region at E14.5. Between E14.5 and E17.5, pendrin expression expands from the hook region to the lower and then to the upper turn of the cochlea. The onset of pendrin expression in the vestibular labyrinth occurs at E16.5. Pendrin in the inner ear functions mainly as a Cl^−^/HCO_3_^−^ exchanger which secretes HCO_3_^−^ into endolymph and thereby elevates the endolymphatic pH [34, 37, 38].

## Development of the inner ear without pendrin

The first mouse model, *Slc26a4*^Δ/Δ^, formerly called *Slc26a4*^−/−^ or *Pds*^−/−^, is a knock-out in which exon 8 of *Slc26a4* is replaced with a neomycin cassette [39]. The replacement introduces a frame shift, which prevents the generation of a functional protein. As in many patients with bi-allelic mutations, *Slc26a4*^Δ/Δ^ mice develop an enlarged vestibular aqueduct and a Mondini-like dysplasia of the cochlea. *Slc26a4*^Δ/Δ^ mice fail to develop hearing and display circling and head bobbing behavior consistent with an overt vestibular defect. The hearing and balance phenotype of *Slc26a4*^Δ/Δ^ mice is more severe than the phenotype in most patients, who are born with residual hearing. Consistent with the recessive inheritance pattern, *Slc26a4*^Δ/+^ mice develop normal sensory systems and normal hearing and balance. *Slc26a4*^Δ/Δ^ and *Slc26a4*^Δ/+^ mice have been used extensively to investigate the consequence of a complete lack of pendrin for the development of the inner ear [6, 25, 26, 34, 37, 38, 40–43]. Several phenotypic features of *Slc26a4*^Δ/Δ^ mice are also observed in a knock-in mouse in which a splice-site mutation is introduced at exon 8 of *Slc26a4* to introduce a frame-shift and a new stop-codon [44].

The first pathobiological alteration of the inner ear of *Slc26a4*^Δ/Δ^ mice is the enlargement of the luminal volume that begins at E14.5 and coincides with cochlear lumen formation [37, 39]. This enlargement appears to be the consequence of an imbalance between fluid secretion and fluid absorption during the growth phase of the inner ear. At E18.5, when the growth phase of the inner ear ends, the enlargement amounts to a ~10-fold larger volume of scala media in the cochlea of *Slc26a4*^Δ/Δ^ mice compared to *Slc26a4*^Δ/+^ mice. This enlargement persists throughout adulthood.

The second pathobiological alteration in the inner ear of *Slc26a4*^Δ/Δ^ mice is the acidification of cochlear endolymph that develops at E15.5 and coincides which the failed onset of pendrin expression [37]. Acidification of the luminal fluid also occurs in the endolymphatic sac and has been documented in the mature inner ear in the cochlea and the utricle of the vestibular labyrinth [34, 38].

Luminal enlargement and acidification are the primary pathobiological alterations, which distribute the effect of pendrin deficiency from pendrin-expressing cells to the entire inner ear [25]. Luminal acidification alters pH-sensitive mechanisms and luminal enlargement limits cell-to-cell communication mechanisms that rely on diffusible factors transmitted via the luminal or abluminal compartment. Impaired cell-to-cell communication may be responsible for the premature onset of connexin 26 expression in basal cells of stria vascularis at E18.5, for the retarded development of the layered structure of stria vascularis at P3 and the retarded development and delayed innervation of the organ of Corti that manifests as a local hypothyroidism between P5–P10 [37, 43].

A remarkable number of secondary consequences of the lack of pendrin expression in the cochlea have been observed and include an increase in the rate of K^+^ secretion by strial marginal cells, oxidative and nitrative stress in stria vascularis, a loss of the K^+^ channel KCNJ10 in intermediate cells, a loss of the endocochlear potential, a rise in the endolymphatic Ca^2+^ concentration, and finally a degeneration of sensory cells and stria vascularis [6, 26, 34, 40, 41]. The increase in the rate of K^+^ secretion is evident from the finding that differences in endolymph K^+^ concentrations between *Slc26a4*^Δ/Δ^ and *Slc26a4*^Δ/+^ mice never exceed a factor of 2, while the volume of endolymph differed by a factor of 10 [25, 26]. Whether the increase in the rate of K^+^ secretion is a function of the enlarged luminal volume or a function of the lower pH is unknown. Marginal cells measure K^+^ concentrations at the apical membrane by an unknown mechanism and low apical K^+^ concentrations lead to an increase in the rate of K^+^ secretion [45, 46]. A pH effect on K^+^ secretion is also conceivable since the K^+^ channel KCNQ1 in the apical membrane of marginal cells is activated by extracellular acidification [47].

Oxidative and nitrative stress is evident from the presence of elevated amounts of nitrated and oxidized proteins in stria vascularis of *Slc26a4*^Δ/Δ^ mice [41]. Oxidative and nitrative stress may be the consequence of higher metabolic rates necessary to maintain higher rates of K^+^ secretion or the consequence of increased free radical production independent of ion transport possibly in the presence of incompletely developed free-radical defense mechanisms. The expression of the K^+^ channel KCNJ10 has been shown to be sensitive to the combination of oxidative and nitrative stress [41]. Loss of KCNJ10 expression is sufficient to abolish the endocochlear potential [48]. It is not known whether tight junctions, which are required for the generation of the endocochlear potential, are compromised by cell stretching and acidification, nor is it known whether junctional compromise contributes to the loss of the endocochlear potential.

Acidification of endolymph is likely a major factor contributing to the elevation of the endolymphatic Ca^2+^ concentration since cochlear epithelial cells express the acid-sensitive Ca^2+^ channels Trpv5 and Trpv6 [34]. The concentration of Ca^2+^ in endolymph of normal mice, such as *Slc26a4*^Δ/+^ mice, is 22 μM, whereas the Ca^2+^ concentration in *Slc26a4*^Δ/Δ^ mice is ~2 mM, which is higher by a factor of ~100 [34]. Whether the endocochlear potential contributes to the level of the endolymphatic Ca^2+^ concentration in *Slc26a4*^Δ/Δ^ and *Slc26a4*^Δ/+^ mice is not known. It is conceivable that the elevated luminal Ca^2+^ concentration is a major factor that contributes to the degeneration of sensory hair cells [39]. Additional factors may be the luminal acidification, local cochlear hypothyroidism and the lack of the endocochlear potential. Similarly, marginal cells of stria vascularis degenerate after P15 and macrophages appear to invade stria vascularis after P30 in *Slc26a4*^Δ/Δ^ mice [40]. Macrophages that accumulate in stria vascularis are strongly pigmented and give stria vascularis a dark appearance [6]. It is unclear whether pigmentation is inherent to macrophages or whether pigmentation is acquired by phagocytosis of melanin granules generated by intermediate cells of stria vascularis.

Secondary consequences of the lack of pendrin expression have also been observed in the vestibular labyrinth and include an increase in the endolymphatic Ca^2+^ concentration, which leads to the formation of giant otoconia [6, 38, 39]. Otoconia in the utricle and saccule of the vestibular labyrinth are normally no larger than ~20 μm. Giant otoconia reach sizes of ~200 μm. Giant otoconia have been reported in *Slc26a4*^Δ/Δ^ mice as well as in *Slc26a4* mutant mice [6, 39, 44, 49]. Similar to the cochlea, the failure to lower the Ca^2+^ concentration in vestibular endolymph may be due to a lack of Ca^2+^ absorption via acid-sensitive Trvpv5 and Trpv6 Ca^2+^ channels [34, 38, 50]. Luminal acidification has been shown to inhibit transepithelial Ca^2+^ reabsorption in epithelial cells of the semicircular canals [38].

## Development of the inner ear with limited pendrin function

The mouse model *Slc26a4^loop/loop^* was identified in a mutagenesis screen for neurosensory disorders and was found to contain a point mutation, S408F, that reduces the anion exchange activity of pendrin without affecting expression [49]. Interestingly, the reduction of pendrin activity results in a phenotype that is similar to the complete loss of pendrin expression seen in *Slc26a4*^Δ/Δ^ mice. *Slc26a4^loop/loop^* mice do not acquire hearing, develop an enlargement of the cochlea, and form giant otoconia in the vestibular labyrinth. This finding points to the importance of sufficient pendrin function during development.

## Development of the inner ear without pendrin expression in the endolymphatic sac

The mouse model *Foxi1*^−/−^ lacks expression of the transcription factor FOXI1, which controls the expression of pendrin in the endolymphatic sac but has no effect on the expression of pendrin in the cochlea or in the vestibular labyrinth [51]. Thus, *Foxi1*^−/−^ mice express pendrin in the cochlea and in the vestibular labyrinth but lack pendrin expression in the endolymphatic sac. The observations that *Foxi1*^−/−^ are deaf, have vestibular dysfunction, and develop prenatally an enlargement of the inner ear point to the importance of the endolymphatic sac for the development of the cochlea and the vestibular labyrinth.

## Development of the inner ear that lacks pendrin expression in the cochlea and the vestibular labyrinth

The mouse model Tg(*B1-hPDS*)*Slc26a4*^Δ/Δ^ was developed to address the question of whether restoration of pendrin solely to the endolymphatic sac is sufficient to rescue inner ear function [52]. The model contains a transgene which consists of the promoter for human *ATP6V1B1* driving the expression of human *SLC26A4*, formerly called *hPDS. ATP6V1B1* codes for the B1-subunit of the H^+^ ATPase, which is expressed in the endolymphatic sac but not in the cochlea or in the vestibular labyrinth [52]. Thus, Tg(*B1-hPDS*)*Slc26a4*^Δ/Δ^ mice express human pendrin in the endolymphatic sac but lack pendrin expression in the cochlea and the vestibular labyrinth. Most interestingly, hearing and balance were fully restored in Tg(*B1- hPDS*)*Slc26a4*^Δ/Δ^ mice. This finding raises the possibility that a spatially limited therapy focused on the endolymphatic sac (a structure that is relatively remote from the cochlea) restores normal hearing and balance.

## Maintenance of hearing without pendrin

A bi-transgenic mouse model, Tg[E], Tg[R]*Slc26a4*^Δ/Δ^, was developed to address the question of whether pendrin is needed for the maintenance of hearing in a fully functional inner ear [53]. This model contains two transgenes, an effector transgene, Tg[E], which consists of the murine promoter of *Slc26a4* that mediates the expression of a transactivator and a responder transgene, Tg[R], which contains a response-element for the doxycycline-bound transactivator to mediate the expression of murine *Slc26a4.* Both transgenes were crossed into the *Slc26a4*^Δ/Δ^ line. In the presence of doxycycline, pendrin was expressed in the inner ear at natural times and natural sites. Omission or withdrawal of doxycycline prohibited expression or led to the rapid cessation of pendrin expression. Thus, pendrin expression could be controlled through doxycycline. Interestingly, loss of pendrin expression in a fully functional inner ear did not affect hearing [53]. This finding demonstrates that pendrin is required for the development but not for the maintenance of hearing.

## Pendrin expression is required during a critical time period during development

The mouse model Tg[E], Tg[R]*Slc26a4*^Δ/Δ^ was used to determine the time period during which pendrin expression is required for normal cochlear development. The critical time period during which pendrin is needed for the development of normal hearing was between E16.5 and P2 [53]. The time period needed for the development of an uncompromised endocochlear potential appears to begin slightly earlier and to last slightly longer. This finding opens the prospect that a temporally limited therapy focused on the prenatal phase of development can restore normal hearing.

## Conclusions

Studies in mouse models have provided tremendous insights into the role of pendrin in inner ear development. Moreover, studies of pendrin-related mouse models have revealed pathobiological mechanisms that may have broad implications beyond hearing loss associated with loss-of-function or hypo-functional mutations of *SLC26A4*. The concept that a temporally and spatially limited therapy may be sufficient to restore normal hearing provides an imperative to develop interventions that secure a life-time of normal hearing in children bearing mutations of *SLC26A4*.

## Figures and Tables

**Fig. 1:**
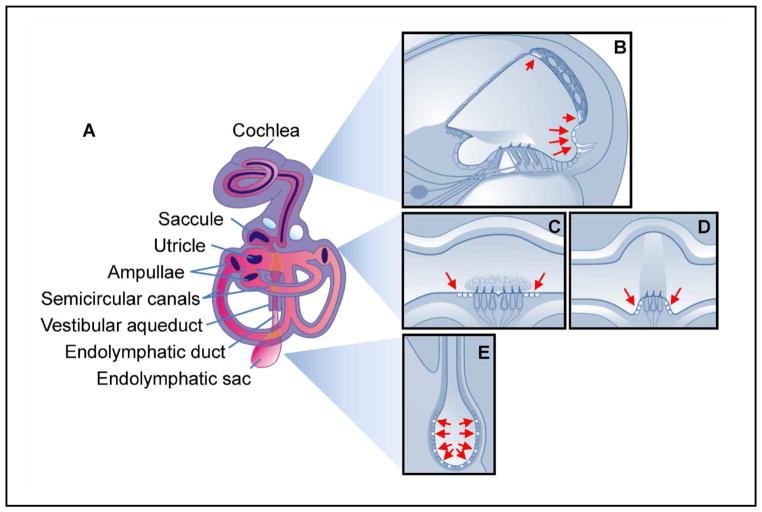
Pendrin expression in the inner ear. A) The inner ear consists of six sensory organs including the cochlea for hearing, the saccule and utricle for sensing linear acceleration and three ampullae and semicircular canals for sensing angular acceleration. In addition, the inner ear contains a non-sensory structure, the endolymphatic duct and sac. All compartments of the inner ear are lined with epithelial cells and filled with endolymph. The epithelial compartments in the cochlea, utricle and saccule, ampullae and semicircular canals are surrounded by perilymph and epithelial compartment of the endolymphatic sac is surrounded by cerebrospinal fluid. B) Cross-section of one turn of the cochlea. The depicted morphology represents the mature stage of development which is acquired in mice during the second postnatal week. Pendrin is expressed in epithelial cells of the spiral prominence (long arrows) and in spindle cells of stria vascularis (short arrows). C–D) Cross-sections of the saccule or utricle and an ampulla. Pendrin is expressed in transitional cells, which are epithelial cells surrounding the maculae in the saccule and utricle and the cupulae in the ampullae. Transitional cells are engaged in cation absorption. E) Cross-sections of the endolymphatic duct and sac. The depicted simple morphology of the endolymphatic sac represents the late phase of embryonic development in the mouse. During the early postnatal period of development, the morphology of the endolymphatic sac becomes more complex with epithelial ridges and tubular infoldings. The endolymphatic duct penetrates through a canal in the bone, which is called the vestibular aqueduct. Pendrin is most prominently expressed in the apical membrane of mitochondria-rich cells in the endolymphatic sac (arrows). A similar diagram was contributed by the author to another paper [52].
